# High Levels of Glyphosate Resistance in *Conyza canadensis* from Agricultural and Non-Agricultural Sites in Ohio and Iowa

**DOI:** 10.1038/s41598-018-28163-w

**Published:** 2018-07-11

**Authors:** Zachery T. Beres, Emily E. Ernst, Bruce A. Ackley, Mark M. Loux, Micheal D. K. Owen, Allison A. Snow

**Affiliations:** 1 Department of Evolution, Ecology, and Organismal Biology, Ohio State University, Columbus, OH 43210 USA; 2 Department of Ecology, Evolution, and Organismal Biology, Iowa State University, Ames, IA 50011 USA; 3 Department of Horticulture and Crop Science, Ohio State University, Columbus, OH 43210 USA; 4 Department of Agronomy, Iowa State University, Ames, IA 50011 USA

## Abstract

Glyphosate is an important herbicide worldwide, but its efficacy has been compromised where weed species have evolved glyphosate resistance. To better understand evolutionary outcomes of continued and strong selection from glyphosate exposure, we characterized variation in resistance in self-pollinating *Conyza canadensis* (horseweed) in Ohio and Iowa, where glyphosate resistance was first reported in 2002 and 2011, respectively. In 2015, we collected seeds from a total of 74 maternal plants (biotypes) from no-till soybean fields *vs*. non-agricultural sites in each state, using one representative plant per site. Young plants from each biotype were sprayed with glyphosate rates of 0x, 1x (840 g ae ha^−1^), 8x, 20x, or 40x. Resistant biotypes with at least 80% survival at each dosage were designated as R1 (1x), R2 (8x), R3 (20x), or R4 (40x). Nearly all Ohio agricultural biotypes were R4, as were 62% of biotypes from the non-agricultural sites. In Iowa, R4 biotypes were clustered in the southeastern soybean fields, where no-till agriculture is more common, and 45% of non-agricultural biotypes were R1–R4. Our results show that resistance levels to glyphosate can be very high (at least 40x) in both states, and that non-agricultural sites likely serve as a refuge for glyphosate-resistant biotypes.

## Introduction

**Figure 1:**
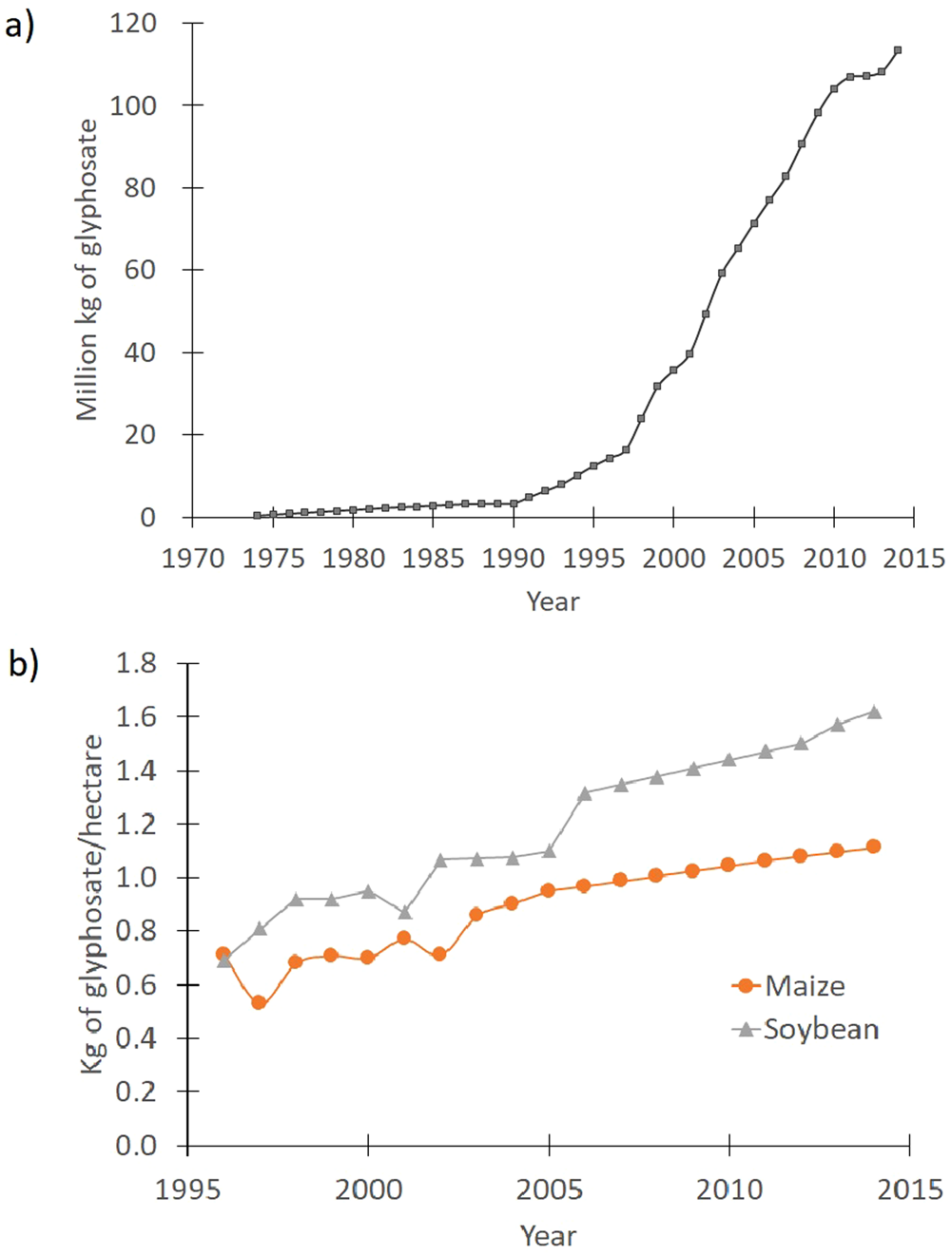
(**a**) Total amount of glyphosate (in kg) used annually in agriculture production in the USA from 1974–2014. (**b**) Amount of glyphosate used per hectare for soybean and maize planted in the USA from 1996–2014. Triangles (▲) correspond to soybean while circles (●) correspond to maize. Data for both (**a**) and (**b**) are from the USDA’s National Agricultural Statistics Service, the Environmental Protection Agency, and the US Geological Survey, as tabulated in the supplemental files of Benbrook 2016. [Benbrook, C.M. Trends in glyphosate herbicide use in the United States and globally. Environmental Sciences Europe. 28, 1–15 (2016)].

The development and ubiquity of transgenic, glyphosate-resistant crops has altered the prevalence and distribution of many agricultural weeds^1,2^. Glyphosate, the active ingredient in Roundup®, is a broad-spectrum, systemic herbicide that inhibits 5-enolpyruvlyshikimate-3-phosphate synthase (**EPSPS; EC 2.5.1.19**), an enzyme in the shikimic acid pathway, thereby blocking the production of three essential aromatic amino acids (tryptophan, tyrosine, and phenylalanine) and other downstream metabolites^3,4^. As a broad-spectrum, low-cost, and highly effective herbicide, glyphosate has become the most commonly used herbicide worldwide, especially in conjunction with transgenic row crops since the late 1990’s^5,6^. The amount of glyphosate used in US agriculture has risen sharply in recent decades (Fig. 1a; Data shown is adapted from supplemental data in Benbrook, C.M. Trends in glyphosate herbicide use in the United States and globally. *Environmental Sciences Europe*. **28**, 1–15 (2016)). In soybean fields, the average amount of glyphosate applied per hectare more than doubled between 1995 and 2015 (Fig. 1b; adapted from Benbrook 2016). By 2016, herbicide-resistant crop varieties in the USA represented 94% of soybean production, 89% of maize, and 89% of cotton^7^, mainly due to transgenic glyphosate resistance. Over-reliance on glyphosate has resulted in the rapid evolution and spread of glyphosate-resistant (GR) weeds. Worldwide, at least 37 weed species are reported to have evolved resistance to glyphosate^8^.

As the abundance and distribution of GR weeds increases over time, growers often use greater herbicide dosages, more frequent applications, and/or use other herbicides and herbicide-tolerant crops with stacked resistance traits^2^. Understanding how quickly high levels of glyphosate resistance evolve and spread has practical applications for weed management and is interesting from an evolutionary biology standpoint. Strong glyphosate selection pressure, multiple origins of glyphosate-resistant biotypes, and gene flow via pollen and seed dispersal have contributed to the current situation in no-tillage agriculture, where the prevalence of GR weed species has greatly complicated weed management and increased production costs^9–11^.

The first case of glyphosate resistance in a broadleaf weed was documented in 2000 in *Conyza canadensis*, also known as horseweed, marestail, or Canada fleabane, after only three years of repeated glyphosate application in Delaware^12^. We chose to focus on this species because GR horseweed is an important weed in no-tillage and low-tillage crops^1,13^. Spring and fall tillage destroy horseweed rosettes, and seeds fail to germinate when buried greater than ~0.5 cm^14,15^. However, the commercialization of Roundup Ready® crops and the concomitant use of glyphosate have facilitated the adoption of no-tillage systems, such that GR horseweed has become very abundant^1,13^. For example, Davis *et al*.^16^ reported widespread glyphosate resistance in Indiana horseweed populations for samples collected from no-till soybean fields in 2003–2005. In California, multiple independent origins of GR horseweed have been documented using genetic markers^17^. GR horseweed now occurs in at least 25 USA states and 12 other countries^8^. Horseweed also has evolved resistance to ALS-inhibitors, paraquat, atrazine, and other herbicides, with some populations evolving resistance to more than one herbicide mode of action^8,18^. Here, we studied horseweed populations in Ohio and Iowa, where GR biotypes were first reported in 2002 and 2011^8^, respectively, and may have been present prior to these reports.

Horseweed is a summer annual or facultative winter annual native to North America, with germination occurring whenever conditions are favorable^19,20^. It often colonizes field margins, roadsides, industrial areas, and other disturbed sites, in addition to row crops, orchards, vineyards, and other perennial crops^21,22^. Rosettes bolt to produce a ~1–2 m tall, multi-branched flowering stem^20,23^. The small, white florets are highly self-pollinating, with about 1–4% outcrossing^24,25^. Individual plants can produce >200,000 tiny, wind-dispersed seeds that exhibit no dormancy and are relatively short-lived in soil seed banks^20,26^. Previous research showed that seeds can disperse >500 km via the upper atmosphere^27^, but Dauer *et al*.^22^ estimated that only ~1% of seeds disperse >100 m from their maternal plants. Nonetheless, seeds from large, heavily infested fields could potentially disperse ~1–5 km per year^22,28^. High levels of seed production and the potential for widespread dispersal have aided in making GR horseweed an economically important agricultural weed. Uncontrolled or poorly managed horseweed populations can form nearly monospecific stands with high population densities and may reduce soybean yields by 90%^29^.

Few studies have documented the full range of evolved resistance levels in GR weed populations. Herbicide resistance studies typically use relatively low discriminating doses such as 1x or 2x of the labeled use rate for a specific herbicide to identify resistant biotypes, and are often supported with dose-response experiments to determine ED_50_ (effective dose for 50% control) or ED_90_ levels for a few representative populations^30,31^. Dose-response studies showed that several GR horseweed biotypes collected in California in 2006 and 2007 were 4.8- to 6-fold more resistant than susceptible plants^21,32^, which is similar to early reports of 4-fold glyphosate resistance in Delaware biotypes^12^. Here, we tested for resistance to as much as 40x the manufacturer’s recommended application rate (1x = 840 g ae ha^−1^) in horseweed populations from regions of Ohio and Iowa where no-tillage soybean production is common. We hypothesized that horseweed would be more resistant to glyphosate in agricultural compared to non-agricultural sites, although seed dispersal from agricultural site populations may introduce GR biotypes into non-agricultural sites.

Unlike most previous studies of GR horseweed, we collected seeds from a single maternal plant from each collection site. This procedure was used because maternal seed families are expected to be full-siblings due to very high selfing rates in horseweed^20,33^, and thus represent evolutionary units on which selection for herbicide resistance can act. In addition, we chose not to use pooled seed samples from several plants per population to avoid mixing seeds from co-occurring horseweed plants that may differ in resistance levels^21,34^. Here, we refer to seeds from the same plant as maternal biotypes (or just biotypes) and we focus on variation in GR at the level of these biotypes rather than populations. The main goals of our study were to determine:

1. The extent to which GR horseweed plants from the study areas are able to tolerate very high dosages of glyphosate application (40x); and
2. The extent to which GR biotypes are present in both non-agricultural sites and agricultural sites when sampled at the end of the growing season.

## Results and Discussion

### High levels of glyphosate resistance

**Figure 2:**
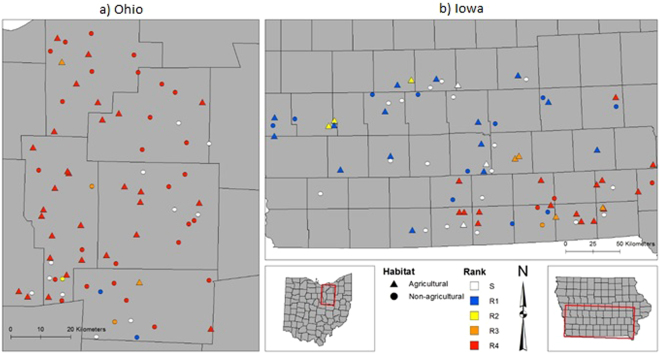
Locations of maternal *Conyza canadensis* (horseweed) biotypes with glyphosate resistance levels and habitats in Ohio (**a**) and Iowa (**b**). Locations with triangles (▲) correspond to biotypes from agricultural habitats and circles (●) correspond to non-agricultural habitats. Biotypes were grouped into glyphosate resistance rankings based on 80% survival at 0x only (S = Susceptible), and up to 1x (R1), 8x (R2), 20x (R3), and 40x (R4). Biotype locations were separated by at least 1.6 kilometers. Maps were generated using ArcGIS® software ver. 10.2.2 for Desktop (Environmental Systems Research Institute Inc., www.esri.com).

**Figure 3:**
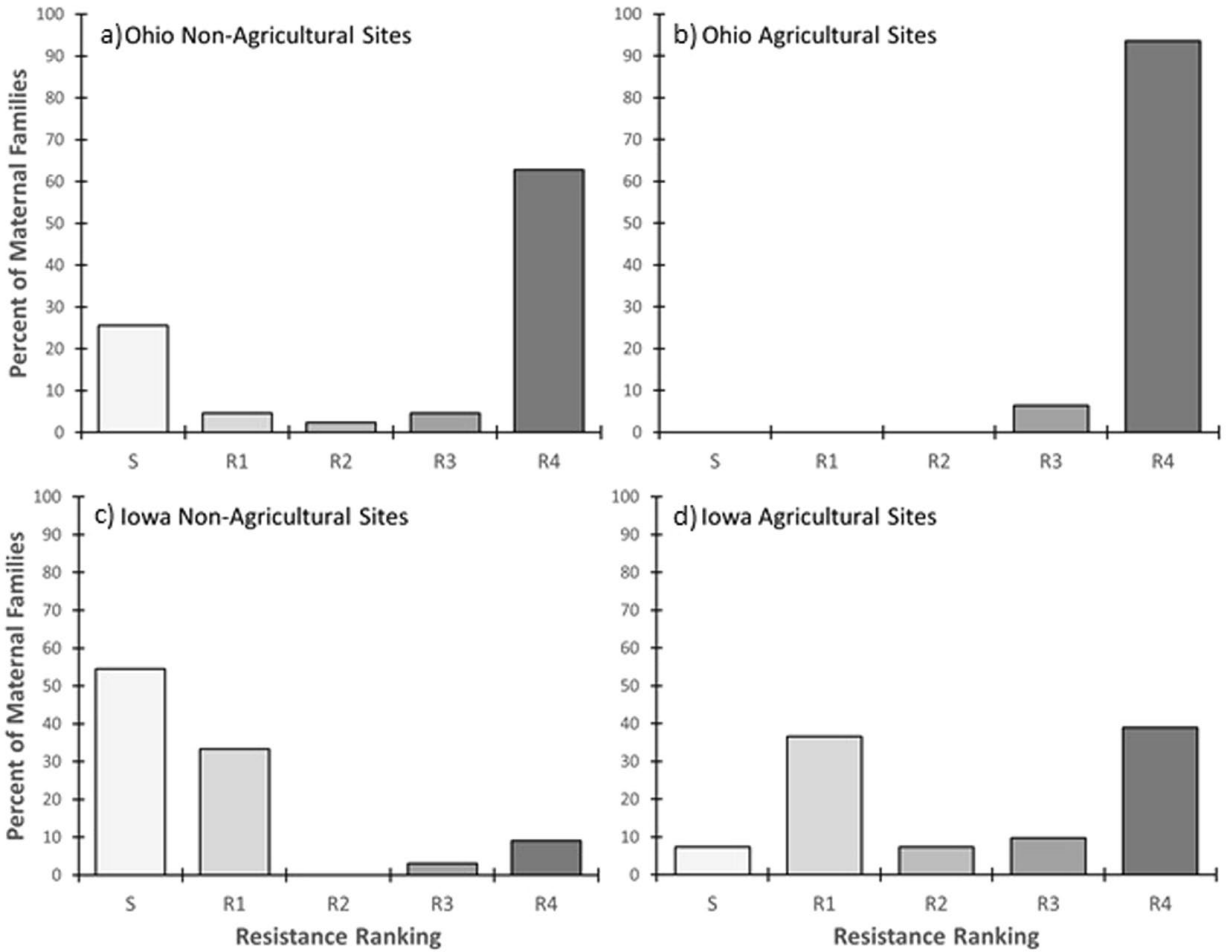
Percent of maternal *Conyza canadensis* (horseweed) biotypes grouped by agricultural habitats and non-agricultural habitats and by glyphosate resistance ranking for Ohio (**a**,**b**) and Iowa (**c**,**d**). Biotypes were grouped into resistance rankings based on 80% survival at 0x only (S = susceptible), and up to 1x (R1), 8x (R2), 20x (R3), and 40x (R4). For Ohio: agricultural habitats (N = 31) and non-agricultural habitats (N = 43). For Iowa: agricultural habitats (N = 41) and non-agricultural habitats (N = 33).

Susceptible biotypes were uncommon among the sampled sites (Figs 2 and 3), as described further below, and a major finding of our study is that extremely resistant R4 biotypes, which had >80% survival at 40x, were found in both states (Figs 2 and 3). To our knowledge, this represents the highest documented levels of GR in horseweed based on discriminating doses. This high level of resistance was very common in samples from Ohio, representing 94% of the sampled biotypes at agricultural sites and 62% at non-agricultural sites, as compared to Iowa, where 39% and 9% of biotypes from agricultural and non-agricultural sites, respectively, were R4. Detection of R4 plants surviving in no-till soybean fields illustrates the challenges that growers face when attempting to manage glyphosate-resistant horseweed, which now requires the use of other herbicide modes of action and/or other control methods such as tillage. Surviving R4 plants are expected to disperse their small, wind-blown seeds within and among neighboring fields, to germinate in the fall or spring on the surface of no-till fields. The prevalence of highly resistant biotypes in Ohio and Iowa may be related to a history of evolved glyphosate resistance dating back to at least 2002 and 2011, respectively^8^. Over time, growers may have employed more frequent and possibly higher dosages of glyphosate applications in an attempt to manage GR horseweed and other species such as GR giant ragweed (*Ambrosia trifida*) in Ohio and Iowa, and GR waterhemp (*Amaranthus tuberculatus*) in Iowa^35–38^ (Fig. 1; adapted from Benbrook 2016).

Combining data from both states, we found that most glyphosate-resistant horseweed biotypes were either R1 (n = 28) or R4 (n = 74; Table 1). It is noteworthy that the majority of the GR biotypes sampled in Ohio were ranked as R4 (76%; n = 56 of 74), while GR biotypes from Iowa had more variable levels of resistance and only 26% (n = 19 of 74) were ranked as R4. Mean visual scores document the relative performance response of surviving R1-R4 biotypes at each glyphosate dosage (Table 1; on a scale of 0 [no damage] to 10 [dead], with 9 being ~90% dead tissue). After the 1x spray, the R1 biotypes maintained healthy, green meristems with new leaves forming and moderate leaf damage that resulted in a mean visual score of 5.5. Extremely resistant R4 plants sustained considerable damage when sprayed with 40x glyphosate (mean score of 6.9), but they maintained healthy, green meristems after treatment and began developing new leaves. We assume that all surviving horseweed were capable of reproduction, similar to what we have observed for drift-damaged GR horseweed plants at the margins of agricultural fields and in our preliminary pilot experiments. Future studies could include greater doses of glyphosate than ours to test for even higher-level resistance, as well as to quantify reproductive capabilities of spray-damaged GR plants.

**Table 1:** Mean visual damage scores for glyphosate-resistant *Conyza canadensis* (horseweed) biotypes.

Dose	Glyphosate resistance rankings							
	R1		R2		R3		R4	
	Mean	(1 SE)	Mean	(1 SE)	Mean	(1 SE)	Mean	(1 SE)
	N = 28		N = 4		N = 9		N = 74	
1x	5.5	(0.4)	3.0	(0.8)	0.9	(0.4)	0.9	(0.1)
8x	—	—	7.4	(0.1)	5.6	(0.3)	4.8	(0.1)
20x	—	—	—	—	6.7	(0.2)	6.1	(0.1)
40x	—	—	—	—	—	—	6.9	(0.1)

Note – Means and 1 Standard Error (SE) are shown based on visual scores at 21 days after application. Biotypes were grouped into resistance rankings based on ≥80% surivial at up to 1x (R1), 8x (R2), 20x (R3), and 40x (R4). Dashes indicate combinations with <80% survival. N indicates the number of maternal biotypes in each category using combined data from Ohio and Iowa. Damage scores ranged from 0 (no damage) to 10 (dead), with 9 being ~90% dead tissue; dead plants were not used in calculations.

### Agricultural *vs*. non-agricultural habitats

Another goal of our study was to characterize variation in levels of glyphosate resistance in two general habitat types – agricultural and non-agricultural – to examine the potential for the latter to serve as a refuge for GR biotypes. At agricultural sites, we collected seeds from mature plants found in no-tillage soybean fields where glyphosate is the most common method of weed control, so these plants were likely exposed to glyphosate earlier in the growing season. In contrast, the non-agricultural sites included roadsides, ditches, residential yards, parks, abandoned fields, and other disturbed sites, where exposure to glyphosate was likely rare or absent.

The frequency of glyphosate-resistant horseweed biotypes was significantly greater for agricultural sites compared to nonagricultural sites (Figs 2 and 3), consistent with expectations, and resistance rankings were not independent of habitat (Ohio: X^2^ = 12.453, d.f. = 4, *P* = 0.0143; Iowa: X^2^ = 24.445, d.f. = 4, P < 0.0001). Only 15% of the sampled horseweed biotypes from Ohio (n = 74) were susceptible to glyphosate, and all of these susceptible seed families were collected from non-agricultural habitats (Fig. 3). In contrast, 7% of the Iowa horseweed biotypes from agricultural habitats were susceptible, while 54% of those from non-agricultural habitats were susceptible (n = 41 and 33 for agricultural and non-agricultural habitats, respectively). Thus, nearly all of the horseweed biotypes from no-tillage soybean fields were resistant to glyphosate, either because any susceptible plants had been killed by the end of the growing season, or because all plants at the site were resistant to glyphosate prior to spraying. For the no-tillage soybean fields in Iowa, the presence of three susceptible biotypes at the end of the growing season suggests that they were not sprayed with glyphosate prior to seed collection, perhaps due to late emergence or variation in herbicide treatments used by growers.

For non-agricultural sites, proximity to farmland where no-till soybean is grown may have contributed to the larger proportion of GR biotypes collected from the sampled sites in Ohio *vs*. Iowa. Many of the non-agricultural sites in Ohio were adjacent to farmland, while more of those in Iowa were located in parks and other semi-natural habitats (Supplementary Table S1). Previous studies also found that GR horseweed occurs in a range of non-agricultural habitats. Gage *et al*.^34^ reported that unsprayed field margins served as a GR horseweed refuge at 17 sites in Illinois and Nebraska. Hanson *et al*.^21^ screened 144 sites for GR horseweed in agricultural field margins *vs*. non-agricultural habitats in 2006 and 2007 in the Central Valley of California. They found high frequencies of GR plants (62% overall) and did not detect correlations with land-use type, presumably because of long-distance seed dispersal from areas where glyphosate applications had selected for GR horseweed biotypes (e.g., in almonds, walnuts, pistachios, grapes, and stone fruit). Our study also found high frequencies of GR horseweed in non-crop habitats, but differences due to habitat type were significant in Ohio and Iowa (Fig. 3). Stronger habitat effects in our study could be due in part to the fact that we sampled plants growing within no-tillage soybean fields whereas Hanson *et al*.^21^ sampled plants from the margins of perennial fruit and nut crops as well as variety of non-soybean annual crops.

### Distribution of GR biotypes in each state

In Ohio, the most resistant horseweed biotypes were evenly spread across the six sampled counties, which are characterized by no-tillage soybean and corn production. Earlier surveys in Ohio found that the prevalence of GR in horseweed decreased from southwestern counties to north-central counties^18^, M. Loux and B. Ackley personal observations. In 2013, we carried out a pilot survey of GR horseweed in soybean fields and non-agricultural sites in southwestern Ohio, and found only one susceptible biotype (data not shown; n = 30 biotypes from three counties). Overall, it appears that GR horseweed has become much more common in Ohio during the past decade, consistent with informal surveys and reports from growers. A notable result from this study is the ubiquity of R4 biotypes in north-central Ohio, making glyphosate nearly useless for managing horseweed in this region and likely other Ohio counties.

In Iowa, the highly resistant R3 and R4 horseweed biotypes were mainly concentrated in the southeast portion of the sampled area (Fig. 2). Horseweed is considered one of the top three problem GR weeds in Iowa, particularly in southern counties were no-tillage production is most common^35^. We expect in the future that continued reliance on glyphosate will increase GR horseweed biotypes throughout Iowa, especially where R1-level resistance has already been found (Fig. 2).

Previous authors have noted the prevalence of GR horseweed in no-tillage Roundup Ready® soybean, where it has become one of the most hard-to-manage weeds^13^. Early warning signs of the extent of this problem came from growers’ observations and surveys such as Davis *et al*.^16^, who sampled horseweed in Indiana soybean fields in 2003–2005. GR horseweed populations were most common in southeastern Indiana, where 78% were classified as resistant and where the highest proportion of cropland was represented by soybean production^16^. Other studies in the USA also have shown that GR horseweed biotypes are more prevalent in southern portions of the sampled area^18,21,34^.

### Mechanisms for the evolution of GR horseweed

Strong selection pressure from glyphosate use has spurred the evolution of multiple mechanisms of glyphosate resistance in a wide range of species e.g.^4,39,40^. Horseweed has been the subject of many studies of glyphosate resistance mechanisms, but little is known about the separate or combined effects of these mechanisms on different levels of resistance (e.g., R1–R4 in this study). The most commonly reported mechanisms for GR in horseweed are reduced translocation and vacuolar sequestration, which have been found in GR horseweed biotypes from Delaware, Ohio, Iowa, Arkansas, Virginia, Mississippi, California, and Greece e.g.^21,41–45^. Vacuolar sequestration is thought to be an active process involving transporter genes that are induced by glyphosate treatment e.g.^46–48^ and may be encoded by a dominant, single-locus nuclear allele^24,25,49^. Ge *et al*.^44^ found evidence for an unknown, “low-level” resistance mechanism for GR in a Delaware biotype, and Gonzalez-Torralva *et al*.^50^ described horseweed biotypes that had reduced translocation and also metabolized glyphosate. Several weed species are resistant to glyphosate due to target site mutations at amino acid 106 of the *epsps* gene^4,51^. A target site mutation conferring strong glyphosate resistance at the 106 locus is widespread in Ontario, Canada^52^, but was not reported for samples from Delaware or Greece^45,48^. Another mechanism that may be present in some horseweed populations is the overproduction of EPSPS^43^, but see ^45,48,53^.

Differences mechanisms found within *Conyza canadensis* may explain the extreme differences in glyphosate resistance that we found among biotypes from Ohio and Iowa. For example, biotypes ranked as R1 may possess a single mechanism such as altered translocation, vacuolar sequestration, or the unknown “low-level” resistance mechanism documented by Ge *et al*.^44^. Conversely, those biotypes ranked as R4 may possess multiple mechanisms, perhaps including a point mutation at the 106 locus. Further research on biotypes with very high levels of GR is needed to understand which mechanisms confer tolerance to glyphosate applications as great as 40x.

## Conclusions

This research focused on maternal biotypes that represented a spectrum of resistance levels in horseweed, rather than biotypes that were simply scored as susceptible *vs*. resistant. It is noteworthy that horseweed populations have evolved very high levels of resistance in both Ohio and Iowa since the earliest documented cases of resistance in 2002 and 2011^8^, respectively. We hypothesize that more than one of several known mechanisms of glyphosate resistance could be present in R4 horseweed biotypes^43,48,50,52^, many of which also could be resistant to ALS inhibitors and other herbicide modes of action^18^. Future studies could include investigations of possible fitness costs that may be associated with various resistance mechanisms in the absence of glyphosate exposure. If no such fitness costs are detected, it seems likely that herbicide-resistant horseweed biotypes could persist indefinitely and contribute to mounting problems for farmers who rely heavily on herbicides for weed management.

## Materials and Methods

### Seed collection

Mature horseweed plants in agricultural habitats (e.g., no-tillage soybean fields which were presumed to have high glyphosate selection pressure) and non-agricultural habitats (presumed to have low glyphosate selection pressure) in Ohio and Iowa were sampled for seeds during fall 2015 (Figs 2 and 3). In choosing where to collect samples, we targeted counties with large areas of soybean production and known horseweed populations. With this sampling method, we did not intend to compare state-wide frequencies of resistant biotypes, and we note that the area over which samples were collected was much greater in Iowa than Ohio (Fig. 2). Collection sites were selected using a targeted (non-random) sampling procedure, with at least 1.6 km between sites.

As noted above, we collected seeds from one mature plant (maternal biotype) at each of the 148 populations. Agricultural site plants were sampled from within the soybean crop unless the crop had been harvested, in which case we sampled from the field edge; harvest had occurred at 16% of the Ohio soybean sites and at none in Iowa. Seeds from each maternal plant were air dried and stored in a paper envelope at 4 C. Sampling resulted in 74 biotypes from six north-central Ohio counties (~3,722 km^2^), and 74 biotypes from 32 southern Iowa counties (~20,135 km^2^; Fig. 2). Sample sizes for the agricultural and non-agricultural sites in each state are shown in Fig. 3. Habitat notes and GPS coordinates for each site are listed in Supplementary Information Table S1.

### Screening for glyphosate resistance

The 148 biotypes were screened for glyphosate resistance in separate greenhouse experiments starting in December 2015 (Iowa biotypes) and March 2016 (Ohio biotypes) using identical protocols. As described below, the randomized complete block design included five concentrations of glyphosate, three trays per biotype at each glyphosate concentration, and 6 plants per tray, for a total of 13,320 plants (5 concentration treatments × 18 plants × 148 biotypes).

Using three planting cohorts separated by a week, seeds from each biotype were germinated in trays (12 cm × 24 cm) with potting soil (Fafard 3B; www.fafard.com) mixed with a slow-release fertilizer (Osmocote, 14-14-14; www.osmocotegarden.com) to minimize nutrient deficiencies, based on the manufacturer’s recommendations for annuals (~360 grams/2.8 ft^3^). Tray positions were randomized weekly to minimize environmental variation in the greenhouse. Greenhouse conditions were 25/18 C day/night with a 14-h photoperiod, and plants were watered as needed. Seedlings were thinned 7 days after planting to leave 6 uniformly-sized and spaced plants within each tray.

When horseweed rosettes were 4–6 cm in diameter, the plants were treated with one of five glyphosate doses: 0x (surfactant and AMS only), 1x (840 g ae ha^−1^; manufacturer’s recommended application rate, which equates to 0.6725% glyphosate (v/v); AquaMaster®, 648 g L^−1^, Monsanto Co.; St. Louis, Misouri), 8x, 20x, or 40x. This rosette size range at application was similar to previous studies of GR horseweed^13,21^. All treatments included ammonium sulfate solution (N-Pak® AMS Liquid, 407 g L^−1^; Winfield Solutions, LLC; St. Paul, Minnesota) and non-ionic surfactant (Preference®; Winfield Solutions, LLC) at 5% and 0.5% (v/v), respectively. Treatments were applied using a pneumatic track sprayer equipped with an even, flat-spray tip (Teejet 8001EVS; Spraying Systems Co.; Carol Stream, Illinois) calibrated to apply 140 L ha^−1^ of spray solution at 3.5 km hr^−1^. Plants were returned to the greenhouse and maintained for 3 weeks as previously described.

At 21 days after treatment, individual plants were visually assessed for leaf damage on a scale from 0 (no damage) to 10 (dead), with 9 being ~90% dead tissue. Proportional survival was calculated for each maternal biotype at each dosage to assign a glyphosate resistance rank. The proportion surviving at each dosage was based on 16–18 plants for 92% of the biotype/treatment combinations, while two biotype/treatments had 4–6 plants, and 26 others had 11–15 plants. Biotypes with less than 80% survival at 1x were classified as “Susceptible.” Resistant biotypes with 80% survival at 1x but less than 80% survival at 8x were classified as “R1.” Likewise, biotypes with >80% survival at 8x were classified as “R2”, >80% survival at 20x as “R3”, and >80% survival at 40x as “R4”. The relative health of resistant survivors 21 days after spraying was determined by calculating the mean visual score for each biotype and the mean across biotypes with the same resistance ranking for each glyphosate concentration (Table 1). Associations between resistance rankings (Susceptible, R1–R4) and habitat (agricultural *vs*. non-agricultural) were tested using Pearson’s X^2^ statistic in R^54^ (Fig. 3; each state was analyzed separately).

## Electronic supplementary material

Supplementary Information
